# A prospective patient registry to monitor safety, effectiveness, and utilisation of bedaquiline in patients with multidrug-resistant tuberculosis in South Korea

**DOI:** 10.1186/s12879-022-07955-6

**Published:** 2023-01-09

**Authors:** Tae Sun Shim, Helen Pai, JeongHa Mok, Seung Heon Lee, Yong-Soo Kwon, Jae Chol Choi, JaeSeok Park, Eileen Birmingham, Gary Mao, Lori Alquier, Kourtney Davis, Florence Thoret-Bauchet, Ji Hyun Kim, Hyeongyeong Kim, Nyasha Bakare

**Affiliations:** 1 Asan Medical Center, University of Ulsan College of Medicine, Seoul, South Korea; 2 Janssen Research and Development, LLC, Raritan, NJ USA; 3 Pusan National University Hospital, Busan, South Korea; 4 Korea University Ansan Hospital, Ansan, South Korea; 5 Chonnam National University Hospital, Chonnam National University Medical School, Gwangju, South Korea; 6 Chung-Ang University Hospital, Seoul, South Korea; 7 Dankook University Hospital, Cheonan, South Korea; 8 Janssen Research and Development, LLC, Titusville, NJ USA; 9 Janssen-Cilag, Issy-Les-Moulineaux, France; 10 Janssen Korea, Seoul, South Korea

**Keywords:** Tuberculosis, Multidrug-resistant, Bedaquiline, South Korea

## Abstract

**Background:**

Multidrug-resistant tuberculosis (MDR-TB) represents a major public health concern, with an ongoing need for new effective treatments. Bedaquiline is an oral diarylquinoline that has shown encouraging treatment success and culture conversion rates in MDR-TB.

**Methods:**

A South Korean patient registry was set up across 19 centres between 2016 and 2018 for the prospective collection of data from patients with MDR-TB who received either a bedaquiline-containing or a non-bedaquiline-containing regimen. Treatment was at the physician’s discretion (bedaquiline use requiring approval by special committee) and was based on patient characteristics, disease status, and local treatment guidelines.

**Results:**

The safety population included 172 patients (88 bedaquiline and 84 non-bedaquiline). The mean (standard deviation, SD) duration of follow-up was 24.3 (9.5) months. Mean (SD) durations of treatment were 5.4 (1.8) months in bedaquiline-treated patients and 15.7 (6.7) months in the non-bedaquiline group. Treatment success (cured and treatment completed according to WHO 2013 treatment outcome definitions) was achieved by 56.3% of bedaquiline-treated and 45.2% of non-bedaquiline-treated patients. Sputum culture conversion rates were 90.4% and 83.7% with and without bedaquiline, respectively. Diarrhoea and nausea were the most frequently reported treatment-emergent adverse events (TEAEs) in the bedaquiline group (27.3% [24/88] and 22.7% [20/88], respectively). The most frequent bedaquiline-related TEAEs were prolonged QT interval (10.2%; 9/88), and diarrhoea and nausea (9.1% each; 8/88). QT interval prolongation was reported in 19.3% (17/88) of bedaquiline-treated and 2.4% (2/84) of non-bedaquiline-treated patients, but bedaquiline was not discontinued for any patient for this reason. There were 13 (14.7%) and three (3.6%) deaths in the bedaquiline-treated and non-bedaquiline groups, respectively. Review of fatal cases revealed no unexpected safety findings, and no deaths were bedaquiline-related. The most common cause of death was worsening cancer (three patients). Patients in the bedaquiline group tended to have poorer baseline risk profiles than non-bedaquiline patients and were more likely to have relapsed or already failed second-line treatment. Interpretation of mortality data was complicated by high rates of loss to follow-up in both groups.

**Conclusions:**

The South Korean registry findings support previous risk/benefit observations and the continued use of bedaquiline as part of combination therapy in patients with MDR-TB.

**Supplementary Information:**

The online version contains supplementary material available at 10.1186/s12879-022-07955-6.

## Introduction

Although an 18% decline was seen in the number of people newly diagnosed with tuberculosis (TB) between 2019 and 2020 (7.1 million to 5.8 million) [1], this is likely caused by health service disruptions linked to the COVID-19 pandemic, and, over the same period, the number of people who died from TB increased from 1.4 million deaths in 2019 to 1.5 million deaths in 2020 [1].

Drug-resistant TB continues to be a critical global public health concern [1]. Globally, the burden of rifampicin-resistant (RR) or multidrug-resistant (MDR: defined as in vitro resistance to isoniazid and rifampicin at minimum) TB is significant, with approximately 3-4% of new cases and 18-21% of previously treated cases of TB being diagnosed as RR- or MDR-TB [1]. World Health Organization (WHO)-reported treatment success was estimated at 59% for patients with MDR/RR-TB in 2018, although this represented a steady increase from 50% in 2012 [1]. South Korea is classified as an intermediate TB burden country, with an annual incidence of 39 cases per 100,000 population [2]. Approximately 3.4% of new patients were diagnosed with RR-TB or MDR-TB in South Korea in 2016, with a 63% treatment success rate [3].

There is a need for new agents to treat MDR-TB, to provide shorter, simpler regimens and improve treatment success rates [1, 4]. Antitubercular drugs include bedaquiline, an oral diarylquinoline with potent bactericidal and sterilising activity that works by inhibiting mycobacterial ATP synthase [5]. Encouraging culture conversion and treatment success rates in adult patients have been shown in phase 2b studies [6, 7] and several observational studies in real-world clinical settings, some of them long term [8-11]. In a retrospective cohort study, adults receiving bedaquiline substitution for second-line injectables (SLIs) in MDR-TB resulted in improved treatment outcomes at 12 months compared with patients who continued to receive SLIs [12]. Other authors report potential for shortening treatment duration with bedaquiline [13], improved health outcomes, and reduced costs when bedaquiline is added to background regimens in countries with high TB burdens [14], and reduced mortality associated with positive treatment outcomes [15, 16].

Bedaquiline has received accelerated or conditional approval for MDR-TB in various countries based on phase 2 data [6, 7, 17, 18]. A multi-country MDR-TB registry (MCR) was originally conceived in 2013 in response to a request from the WHO [19], the US Food and Drug Administration (FDA), and health authorities in other countries for additional real-world data on the use of bedaquiline in the treatment of MDR-TB, to support continued implementation of bedaquiline-based combination therapy in MDR-TB. This request was based on phase 2 mortality findings, even though no causal patterns were evident [6]. Moreover, evidence that has emerged since accelerated approval has led to the updating of WHO treatment guidelines, which now recommend bedaquiline as a Group A medicine to be prioritised in longer treatment regimens and for use in standardised all-oral shorter regimens [20].

Two countries were selected to provide data for the MCR: South Africa (retrospective collection from a national electronic drug-resistant TB registry) and South Korea (prospective collection from 19 sites). The present paper reports registry findings for South Korea, where the MDR-TB patient registry was implemented as a post-marketing safety study specifically to meet the post-marketing requirement for bedaquiline from the South Korea Ministry of Food and Drug Safety. As registry designs and data collection methods differed between countries, pooled analysis was not possible, and data for South Africa will be reported elsewhere.

This analysis of the prospective MDR-TB registry in South Korea aims to further assess the benefits and risks of bedaquiline by evaluating safety, effectiveness, and emergence of resistance, drug utilisation, and adherence to WHO guidance on the use of bedaquiline in MDR-TB treatment in clinical practice.

## Methods

### The South Korea registry

The registry was implemented across 19 sites (16 medical centres and three national public health centres) with an enrolment target of 60 patients per group (bedaquiline-treated and non-bedaquiline-treated) between 2016 and 2018. During the registry period, treatment was based on the five-group MDR-TB drug classification derived from WHO 2014 guidelines [21], in which bedaquiline was included in a category of drugs not for use in standard regimens because of the limited availability of data relating to efficacy or long-term safety.

The use of bedaquiline was regulated by the National TB Expert Review Committee (NTBERC) of the Korean Centers for Disease Control and Prevention (KCDC) (see Hwang et al. [22]), dating from September 2016 and applied throughout the study period. Bedaquiline was to be used only if approved by a committee of TB experts established under the KCDC. Treatment with either a bedaquiline-containing or a non-bedaquiline-containing MDR-TB regimen was at the treating physician’s discretion (following approval by committee if necessary), and was based on patient characteristics, disease status, and local treatment guidelines (Additional file 1). The main criterion for use of bedaquiline was ‘*an effective regimen cannot be composed from other conventional drugs*’, usually encountered in patients with pre-extensively drug-resistant TB (pre-XDR-TB; defined as MDR plus resistance to fluroquinolones or at least one of the three SLIs amikacin, kanamycin, and capreomycin) or XDR-TB (defined as MDR plus resistance to fluroquinolones and at least one of the three SLIs).

### Study design and patients

Relevant regulatory and ethical approvals and informed consent from patients were obtained. Eligible patients were those with newly diagnosed MDR-TB, including those newly treated with bedaquiline. There were no specific exclusion criteria (i.e., patients could have been previously treated for TB). Patients with pre-XDR-TB or XDR-TB and those with simple MDR-TB with pyrazinamide resistance or simple MDR-TB with resistance to more than two secondary drugs were eligible.

In this non-randomised observational study, choice of MDR-TB regimen (bedaquiline- or a non-bedaquiline-containing) was at the treating physician’s discretion, with bedaquiline use requiring approval by special committee. The total registry treatment and follow-up period was up to 30 months: 18-24 months’ MDR-TB therapy, including bedaquiline for up to 6 months as per label if prescribed, with an additional 6 months of registry follow-up after MDR-TB treatment completion (unless patients were lost to follow-up or died).

### Data collection

A database and electronic clinical report form (eCRF) were created for the purposes of the registry. Data were collected at each site as per routine clinical practice and collated in the Oracle Remote Data Capture (OC-RDC) onsite system (v4.6.6) developed for the registry and maintained by a contract research organisation (Syneos Health).

Adverse events (AEs) were collected according to the WHO guidance for pharmacovigilance of medicines used in the treatment of TB, current at the time of the registry [23]. All AEs and treatment-emergent AEs (TEAEs) were recorded, including those that were serious or bedaquiline-related, or that led to withdrawal, interruption, or dose reduction of bedaquiline, and deaths and events leading to death.

AEs of special interest (AESIs) were identified from the known clinical safety profile of bedaquiline and preclinical data, and included, per Medical Dictionary for Regulatory Activities (MedDRA) version 17, Acute Pancreatitis (Standardised MedDRA Query [SMQ]), Rhabdomyolysis/Myopathy (SMQ), Torsades de Pointes/QT Prolongation (SMQ), and Drug-related Hepatic Disorders (SMQ). Electrocardiographic (ECG) data were collected to monitor QT prolongation, with QT interval defined with correction using Fridericia’s formula (QTcF).

Effectiveness/clinical response was assessed in terms of treatment outcome (cured, treatment completed, died, treatment failed, lost to follow-up) as per 2013 WHO guidance [19], stratified by drug resistance type; sputum culture conversion, defined as two consecutive negative sputum cultures taken at least 30 days apart; and median time to initial sputum culture conversion. Data relating to dosages, duration, treatment compliance/adherence, treatment history, medical history, and concomitant medications used for conditions other than TB were collected.

Baseline resistance profiles were defined per the 2013 WHO guidance [19]:

- MDR-TB excluding pre-XDR and XDR (resistant to isoniazid and rifampicin: MDR-TB_H&R_);
- pre-XDR-TB due to fluoroquinolones (pre-XDR-TB_FQ_);
- pre-XDR-TB due to SLIs (pre-XDR-TB_SLI_);
- XDR-TB (resistance to isoniazid, rifampicin, fluoroquinolones, and SLIs).

Bedaquiline drug susceptibility testing (DST) data were not collected systematically, although DSTs are performed in most culture-confirmed patients in South Korea. Limited DST data (proportions of patients with isolates resistant at baseline) for other MDR-TB drugs only were available for analysis. Concomitant medications of interest were other anti-TB drugs, antidiabetic drugs, antidepressants, cardiovascular medications, and antiretroviral treatment.

### Data analysis

Three analysis populations were defined: the enrolled population; the safety population of all patients exposed to at least one dose of study treatment; and the evaluable population. The evaluable study population, assessed for treatment success/failure, consisted of those patients who had been exposed to at least one dose of study treatment, had a baseline assessment, and had at least one post-baseline assessment. Accordingly, patients with no baseline assessment were excluded from the analysis of treatment success but were still assessed for safety.

Mortality was analysed descriptively as deaths per WHO treatment outcome definition. AEs and TEAEs were summarised descriptively and presented by preferred term. AESIs were grouped by SMQ. Laboratory and ECG data were analysed descriptively.

Prescribing and utilisation of bedaquiline (including indication for bedaquiline use) were analysed descriptively to show total dose and duration, mean with standard deviation (SD) and median dosages, and utilisation of MDR-TB treatments, including duration on-treatment. Frequency distributions with proportions and 95% confidence intervals (CIs) of WHO treatment outcome (cured, treatment completed, treatment failed, lost to follow-up, not evaluated, and treatment success) for bedaquiline-treated and non-bedaquiline-treated patients were prepared.

Frequency distributions of sputum culture conversion were summarised, and the median time to initial sputum culture conversion was estimated using the Kaplan-Meier method. Proportions of patients with isolates resistant to TB drugs (except for bedaquiline) at baseline were summarised. (Additional file 2)

## Results

### Study population

#### Patient disposition

Data were available for all 172 enrolled patients with MDR-TB. The first patient was enrolled on 3 May, 2016, and the last contact date listed in the clinical database was 1 July, 2020. Eighty-eight patients received a bedaquiline-containing MDR regimen and 84 received a non-bedaquiline-containing MDR regimen (safety population); 171 patients had baseline and at least one post-baseline and/or microbiological assessments (evaluable population) and were therefore assessed for WHO treatment outcomes. Approximately half (51.2%) of patients were hospitalised at the time of treatment.

In the evaluable population, 61.4% (54/87) of bedaquiline-treated and 54.8% (46/84) of non-bedaquiline-treated patients completed the study; Table 1 describes patients lost to follow-up, those that died, and patients who withdrew from treatment.

**Table 1:** Baseline demographics and characteristics: safety population

Characteristic, n (%), unless stated otherwise	Bedaquiline N = 88	No bedaquiline N = 84
Mean age, years (SD)	48.6 (15.4)	50.1 (17.9)
Age category, years		
≥ 65	12 (13.6)	18 (21.4)
18-64	76 (86.4)	66 (78.6)
Sex		
Male	57 (64.8)	54 (64.3)
Female	31 (35.2)	30 (35.7)
Resistance category^a^		
MDR-TB	60 (68.2)	78 (92.9)
Pre-XDR-TB	5 (5.7)	1 (1.2)
XDR-TB	23 (26.1)	5 (6.0)
Patient treatment category		
New	31 (35.2)	49 (58.3)
Relapsed	28 (31.8)	17 (20.2)
Treatment after loss to follow-up	4 (4.5)	2 (2.4)
Treatment after failure 1st-line drugs	13 (14.8)	14 (16.7)
Treatment after failure 2nd-line drugs	12 (13.6)	2 (2.4)
Indication for MDR-TB treatment		
Pulmonary TB	86 (97.7)	80 (95.2)
Extra-pulmonary TB	0	4 (4.8)
Missing	2 (2.3)	0
Evidence of cavitary disease on CXR or CT		
Yes	51 (58.0)	42 (50.0)
No	34 (38.6)	34 (40.5)
Uncertain	3 (3.4)	8 (9.5)

*CT* computed tomography, *CXR* chest x-ray, *MDR* multidrug-resistant, *SD* standard deviation, *TB* tuberculosis, *XDR* extensively drug-resistant

^a^Investigator assigned (not based on drug susceptibility testing)

The mean (SD) duration of registry follow-up was 24.3 (9.5) months overall: 24.1 (8.8) months for the bedaquiline group and 24.4 (10.3) months for the non-bedaquiline group. The total person-time of follow-up was 177.1 person-years for patients on a bedaquiline-containing regimen and 170.5 person-years for those on a non-bedaquiline-containing regimen.

#### Baseline demographics and characteristics

Patients’ baseline demographics and characteristics are shown in Table 1. Among the 88 bedaquiline-treated and 84 non-bedaquiline-treated patients, respectively: 60 (68.2%) and 78 (92.9%) had MDR-TB; 28 (31.8%) and 6 (7.1%) had pre-XDR-TB or XDR-TB.

Because treatment selection was at the physicians’ discretion and treatment with a bedaquiline regimen was subject to approval by committee in South Korea, there were several notable differences between the two treatment groups. Key differences in the bedaquiline group compared with the non-bedaquiline group were younger age (86.6% vs, 78.6% were < 65 years, respectively), wider ranging drug resistance (26.1% vs, 6.0%, respectively, had XDR-TB; as assigned by the investigator), failure on second-line therapy (13.6% vs, 2.4%, respectively), and evidence of cavitary disease (58.0% vs, 50.0%, respectively).

#### Treatment information and bedaquiline drug utilisation

Of 88 bedaquiline-treated patients, 83 (94.3%) received bedaquiline for up to 6 months. The mean (SD) total dose was 21,893 (19,881) mg, ranging from 400 mg to 192,800 mg. Mean (SD) durations of bedaquiline treatment and overall TB treatment in bedaquiline-treated patients were 5.4 (1.8) months and 15.7 (6.7) months, respectively.

For bedaquiline-treated patients, compliance was checked by direct observation of treatment for 29/88 (33.0%) patients, nurse call for 21/88 (23.9%), and pill counting for 14/88 (15.9%) (assessment by more than one method was possible). For the three methods, respectively, adherence (defined as taking > 80% of prescribed doses) was 89.7% (26/29 patients), 85.7%, (18/21), and 100% (14/14).

Other anti-TB drugs used in more than 50% of patients are listed in Additional file 1: Table S1. Of the 88 patients who received bedaquiline, 68 (77.3%) received cycloserine, 63 (71.6%) linezolid, and 62 prothionamide (70.5%). Of the 84 non-bedaquiline-treated patients, 75 (89.3%) received prothionamide, 74 (88.1%) cycloserine, 72 (85.7%) pyrazinamide, 59 (70.2%) kanamycin, and 44 (52.4%) levofloxacin.

#### Concomitant medications

Use of any concomitant medication during MDR-TB treatment was reported for all 88 bedaquiline-treated patients and 79/84 (94%) non-bedaquiline-treated patients. The use of concomitant medication in selected classes during MDR-TB treatment was reported in 76/172 (44.2%) patients overall and included cardiovascular drugs in 55 (32.0%), antidiabetics in 26 (15.1%), antidepressants in 18 (10.5%), and antivirals in five (2.9%).

#### Baseline drug susceptibility

Routinely collected baseline DST data for other MDR-TB drugs were as follows for 155 patients overall, and for 84 bedaquiline-treated and 71 non-bedaquiline-treated patients:

- MDR-TB_H&R_: 81 (52.3%), 24 (28.6%), and 57 (80.3%);
- pre-XDR-TB_FQ_: 36 (23.2%), 32 (38.1%), and 4 (5.6%);
- pre-XDR-TB_SLI_: 7 (4.5%), 5 (6.0%), and 2 (2.8%);
- XDR-TB: 21 (13.5%), 17 (20.2%), and 4 (5.6%).

Baseline DST results suggested a greater proportion of patients with severe resistance profiles (pre-XDR-TB or XDR-TB) in the bedaquiline-treated group than did patients’ baseline characteristics. The discrepancy may have been due to differences in accuracy of testing between the reference laboratory (phenotypic DST) and local laboratories (rapid DST based on molecular tests such as Xpert^®^ MTB/RIF, Cepheid, Sunnyvale, CA, USA, or line probe assay).

### Bedaquiline effectiveness

#### WHO treatment outcome

Treatment success was reported in 49/87 (56.3%) bedaquiline-treated and 38/84 (45.2%) non-bedaquiline-treated patients; 37 (42.5%) and 23 (27.4%), respectively, were classified as ‘cured’ (Table 2). Treatment was completed in 12 (13.8%) bedaquiline-treated and 15 (17.9%) non-bedaquiline-treated patients (Table 2). Thirteen (14.9%) and three patients (3.6%) died in the bedaquiline- and non-bedaquiline-treated groups, respectively (Table 2). Numbers of patients lost to follow up were 20/87 (23.0%) and 32/84 (38.1%) in the bedaquiline-treated and non-bedaquiline-treated groups, respectively.

**Table 2:** WHO treatment outcomes and microbiologic outcomes: evaluable population

WHO outcomes, n (%), and [95% CIs]	Bedaquiline N = 87	No bedaquiline N = 84
Cured	37 (42.5) [32.0, 53.6]	23 (27.4) [18.2, 38.2]
Treatment completed	12 (13.8) [7.3, 22.9]	15 (17.9) [10.4, 27.7]
Died	13 (14.9) [8.2, 24.2]	3 (3.6) [0.7, 10.1]
Lost to follow-up	20 (23.0) [14.6, 33.2]	32 (38.1) [27.7, 49.3]
Treatment failed	0 (0) [0.0, 4.2]	1 (1.2) [0.0, 6.5]
Not evaluated	5 (5.7) [1.9, 12.9]	7 (8.3) [3.4, 16.4]
Other	0 (0) [0.0, 4.2]	3 (3.6) [0.7, 10.1]
Treatment success (cured + treatment completed)	49 (56.3) [45.3, 66.9]	38 (45.2) [34.3, 56.5]
Microbiologic results, n (%)		
Sputum culture at baseline		
Negative	26 (29.9)	20 (23.8)
Positive	52 (59.8)	49 (58.3)
Contaminated	2 (2.3)	1 (1.2)
Missing	7 (8.0)	14 (16.7)
Sputum culture at end-of-treatment		
Negative	82 (95.3)	74 (90.2)
Positive	1 (1.2)	6 (7.3)
Contaminated	1 (1.2)	0
Other	2 (2.3)	2 (2.4)
Sputum culture conversion^a^		
To negative	47/52 (90.4)	41/49 (83.7)
Non-conversion	5/52 (9.6)	8/49 (16.3)
Sputum culture reversion		
To negative	2/47 (4.3)	1/41 (2.4)
Non-reversion	45/47 (95.7)	40/41 (97.6)
Times to sputum culture initial conversion	N = 52	N = 49
Median (days)	39	50
95% CI	28, 54	35, 64

*CI* confidence interval, *WHO* World Health Organization; The 95% CI is based on the Clopper-Pearson exact binomial method

^a^Culture conversion defined as two consecutive negative sputum results taken at least 30 days apart

#### Sputum culture results

Positive sputum cultures were reported at baseline in 52/87 (59.8%) bedaquiline-treated and 49/84 (58.3%) non-bedaquiline-treated patients (Table 2). Of these, 47/52 (90.4%) and 41/49 (83.7%), respectively, achieved culture conversion; respective median times to culture conversion were 39 and 50 days in the bedaquiline and non-bedaquiline groups (Table 2).

Three patients, two bedaquiline-treated and one non-bedaquiline-treated, had culture reversion. In the bedaquiline group, one patient achieved culture conversion after 94 days and reverted to positive 34 days later. This patient completed follow-up, and the WHO outcome was recorded as not evaluated. The other patient achieved culture conversion after 15 days and reverted to positive 254 days later; the WHO outcome was recorded as lost to follow-up. The non-bedaquiline-treated patient achieved culture conversion after 352 days and reverted to positive 24 days later, with a WHO outcome of lost to follow-up.

### Safety outcomes

The most frequent TEAEs are shown in Table 3. TEAEs reported in ≥ 20% of bedaquiline-treated patients were diarrhoea (24/88 patients; 27.3%) and nausea (20/88; 22.7%). TEAEs in ≥ 20% of non-bedaquiline-treated patients were nausea (27/84; 32.1%) and arthralgia (24/84 (28.6%). TEAEs in ≥ 5% of patients in either group included diarrhoea (27.3% and 10.7% in bedaquiline and non-bedaquiline-treated patients, respectively), prolonged QT interval (19.3% and 2.4%, respectively), dyspnoea (15.9% and 6%), productive cough (10.2% and 4.8%), hypotension (5.7% and 0%), peripheral neuropathy (13.6% and 2.4%), and paraesthesia (17% and 8.3%).

**Table 3:** Summary of treatment-emergent adverse events (TEAEs): safety population

Parameter, n (%)	Bedaquiline N = 88	No bedaquiline N = 84
≥ 1 TEAE	84 (95.5)	76 (90.5)
≥ 1 bedaquiline-related TEAE	35 (39.8)	NA
≥ 1 TEAE leading to bedaquiline withdrawal	2 (2.3)	NA
≥ 1 serious TEAE	42 (47.7)	27 (32.1)
≥ 1 TEAE leading to death^a^	12 (13.6)	3 (3.6)
TEAEs by preferred term reported in > 10% of patients overall		
Nausea	20 (22.7)	27 (32.1)
Arthralgia	16 (18.2)	24 (28.6)
Diarrhoea^b^	24 (27.3)	9 (10.7)
Cough	16 (18.2)	12 (14.3)
Pruritus	15 (17.0)	12 (14.3)
Decreased appetite	12 (13.6)	15 (17.9)
Dyspepsia	9 (10.2)	15 (17.9)
Dizziness	13 (14.8)	11 (13.1)
Paresthaesia^b^	15 (17.0)	7 (8.3)
Headache	7 (8.0)	13 (15.5)
Rash	8 (9.1)	12 (14.3)
Dyspnoea^b^	14 (15.9)	5 (6.0)
Electrocardiogram QT prolonged^b^	17 (19.3)	2 (2.4)
Anaemia	10 (11.4)	8 (9.5)

*NA* not available

^a^Based on adverse event (AE) reporting, 12 and 3 patients were reported with one or more fatal AEs. One additional fatal case was reported in the bedaquiline-treated group based on efforts to confirm the vital status of patients who were previously reported as lost to follow-up

^b^Reported more frequently in bedaquiline-treated patients (> 5% difference)

Most frequent bedaquiline-related TEAEs (≥ 5.0% of patients) were prolonged QT (10.2%), and diarrhoea and nausea (9.1% each) (Additional file 1: Table S2). Two bedaquiline-treated patients had TEAEs leading to treatment withdrawal, all of which were considered possibly related to bedaquiline by the investigator (Table 3). One patient experienced moderately increased aspartate aminotransferase and alanine aminotransferase and mild dyspepsia; the other had moderate drug-induced liver injury.

AESIs were reported in both groups. AEs from the Acute Pancreatitis SMQ were reported in a single patient (in this case it was acute pancreatitis). AEs from the Torsade de Pointes/QT Prolongation SMQ were reported in 17/88 (19.3%) bedaquiline-treated and 3/84 (3.6%) non-bedaquiline-treated patients. All events were QT prolongation, except for serious cardiac arrest that resolved on the same day in a single non-bedaquiline-treated patient. This patient had co-existing pneumonia and died from aspiration 3 months later. There were no reported cases of Torsades de Pointes. Nineteen (21.6%) bedaquiline-treated and 15 (17.9%) non-bedaquiline-treated patients had Drug-related Hepatic Disorders (SMQ); these were judged as drug-related liver injuries in five and seven patients, respectively. No Rhabdomyolysis/Myopathy (SMQ) events were reported.

Serious TEAEs were reported in 42 (47.7%) and 27 (32.1%) patients treated with and without bedaquiline, respectively (Tables 3 and 4). The most frequently reported serious TEAEs in bedaquiline-treated patients (≥ 2.5%) were dyspnoea and pneumonia (5.7% each), and TB (reported as a TEAE), asthenia, peripheral oedema, and prolonged QT (3.4% each; Table 4). Drug-induced liver injury and acute renal failure (3.6% each) were reported mainly in patients not receiving bedaquiline (Table 4).

**Table 4:** Summary of serious and fatal treatment-emergent adverse events (TEAEs): safety population

Parameter, n (%), unless stated otherwise	Bedaquiline N = 88	No bedaquiline N = 84
Patients with ≥ 1 serious TEAE	42 (47.7)	27 (32.1)
Serious TEAEs by preferred term reported in > 1 patient overall		
Dyspnoea	5 (5.7)	1 (1.2)
Pneumonia	5 (5.7)	1 (1.2)
Asthenia	3 (3.4)	2 (2.4)
Peripheral oedema	3 (3.4)	1 (1.2)
Acute renal failure	1 (1.1)	3 (3.6)
Tuberculosis	3 (3.4)	0
Electrocardiogram QT prolonged	3 (3.4)	0
Chest pain	2 (2.3)	1 (1.2)
Drug-induced liver injury	0	3 (3.6)
Paraesthesia	2 (2.3)	0
Ascites	2 (2.3)	0
Nausea	2 (2.3)	0
Delirium	2 (2.3)	0
Hypotension	2 (2.3)	0
Anaemia	2 (2.3)	0
Optic neuropathy	2 (2.3)	0
Cough	1 (1.1)	1 (1.2)
Peripheral neuropathy	1 (1.1)	1 (1.2)
Depressed mood	1 (1.1)	1 (1.2)
Arthralgia	1 (1.1)	1 (1.2)
Rash	1 (1.1)	1 (1.2)
Influenza	0	2 (2.4)

*MDR* multidrug-resistant, *SD* standard deviation, *TB* tuberculosis, *XDR* extensively drug-resistant

**Fig. 1:**
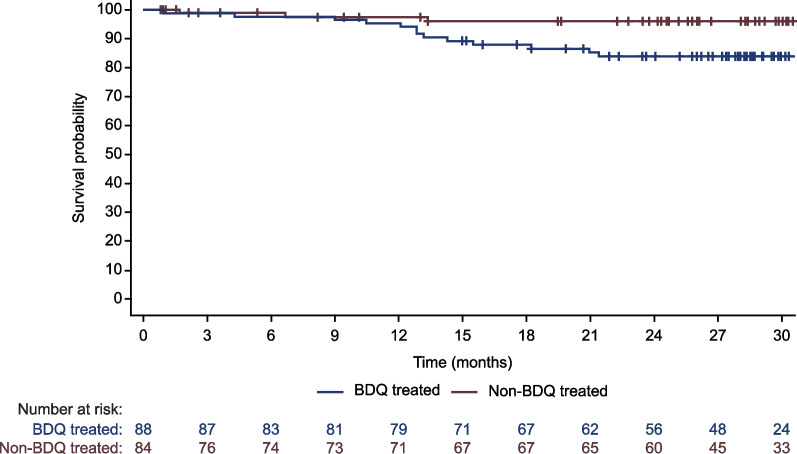
Kaplan-Meier plot of overall survival in South Korean registry patients

Twelve bedaquiline-treated and three non-bedaquiline-treated patients had a TEAE leading to death (Tables 3, 4**,** and Additional file 1: Table S3; Fig. 1). Following efforts to confirm the vital status of patients previously reported as lost to follow-up, one additional fatal case was also reported in the bedaquiline-treated group (Table 3). Seven of the above 13 bedaquiline-treated patients and all three non-bedaquiline-treated patients died while on or within 2 weeks of stopping MDR-TB treatment. All TEAEs leading to death except for pneumonia (five patients) and dyspnoea (three patients) were reported in a single bedaquiline-treated patient (Table 4).

The most frequently reported laboratory abnormality was abnormal haemoglobin levels, observed in 17 (19.3%) bedaquiline-treated and 19 (22.9%) non-bedaquiline-treated patients.

**Fig. 2:**
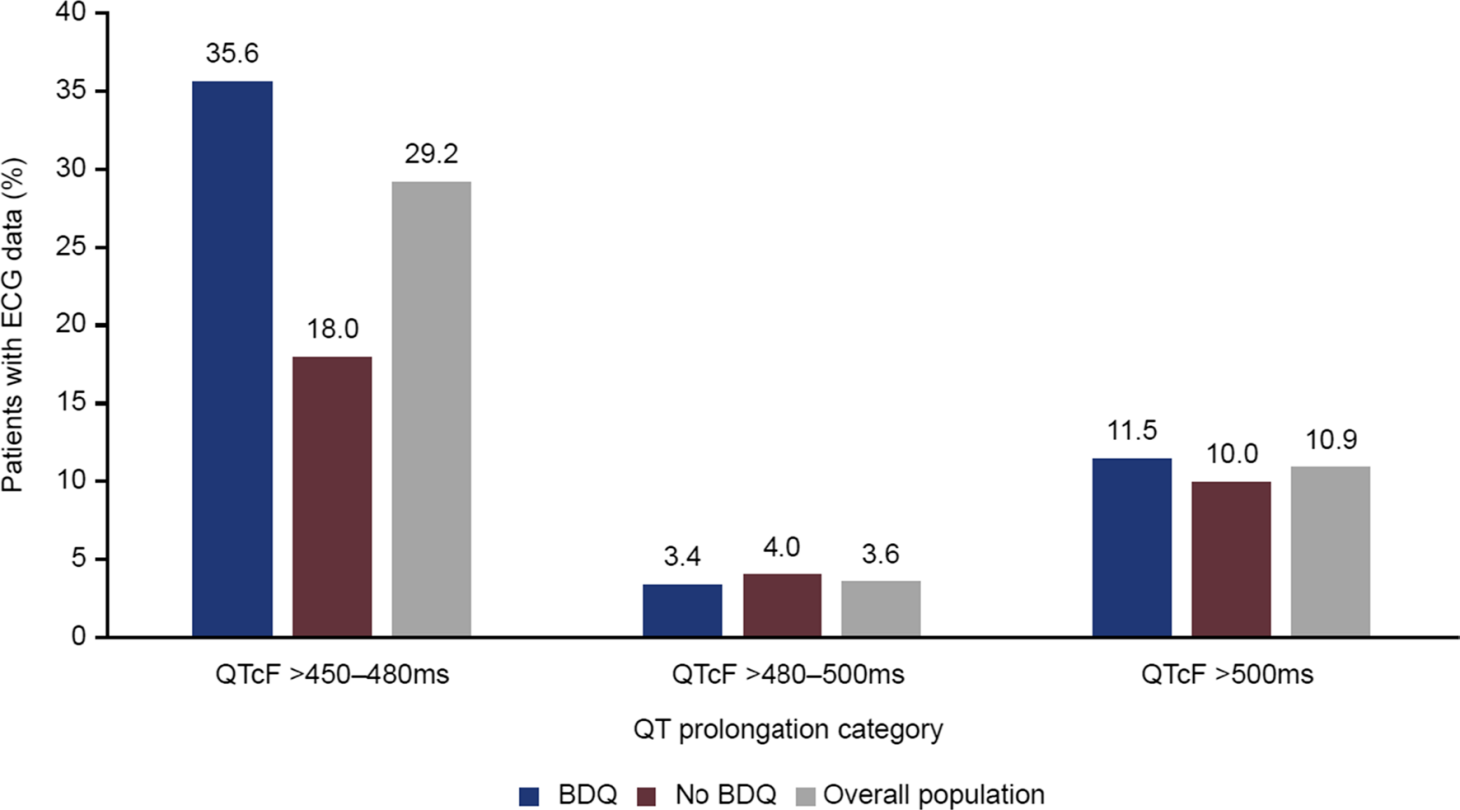
Post-baseline electrocardiogram (ECG) data (available for 87 bedaquiline-treated and 50 non-bedaquiline-treated patients)

ECG results are summarised in Fig. 2. As a result of monitoring requirements [24], ECGs were recorded routinely in bedaquiline-treated patients only; thus, more ECG data were available from the bedaquiline-treated group. Abnormal QTcF values (> 480-500 ms) were reported in 3/87 (3.4%) and 2/50 (4.0%) patients in the respective groups; QTcF > 500 ms was observed in 10/87 (11.5%) and 5/50 (10.0%) patients, all of whom were receiving at least one other drug associated with QT prolongation. Of the 10 bedaquiline-treated patients, six had received clofazimine, five moxifloxacin, and four levofloxacin. There were two treatment interruptions but no permanent discontinuations of bedaquiline as a result of QT prolongation.

## Discussion

Treatment success (sum of cured and treatment completed according to WHO 2013 treatment outcome definitions) was achieved by 56.3% of bedaquiline-treated and 45.2% of non-bedaquiline-treated patients included in this South Korean registry. Serious TEAEs were seen in 47.7% and 32.1% of patients, and death (defined as a WHO treatment outcome) was reported in 14.9% and 3.6% of patients, respectively. Note that 23.0% of bedaquiline-treated patients and 38.1% of non-bedaquiline-treated patients were lost to follow-up, and vital status could not be confirmed for all patients at the end of the study. These factors limit the assessment and interpretation of long-term mortality. Bedaquiline was given for ≤ 6 months, consistent with approved treatment recommendations [25], in 94% of patients. Background regimen drugs were also in line with local guidelines. No new safety signals were identified for bedaquiline based on the AE data.

Although there were more deaths in the bedaquiline-treated group (14.9%) than in the group not receiving bedaquiline (3.9%), the review of fatal cases revealed no unexpected safety findings, and no deaths were considered by investigators to be related to bedaquiline. Eight of the deaths in the bedaquiline group were reported more than 6 months after stopping bedaquiline treatment and included four deaths due to underlying malignancy. Notably, the mortality rates in bedaquiline-treated patients were similar to those reported in a previous national registry study conducted in South Korea between 2011 and 2015 (9.7-11.9% in patients with pre-XDR-TB and 16.4% in patients with XDR-TB) [26].

This was an observational study where treatment allocation was not randomised; consequently, patients were likely channelled to treatment based on their demographic and clinical characteristics, resulting in an imbalance in baseline risk factors between the two treatment groups. In addition, use of bedaquiline in this South Korea registry was regulated by the pre-review system, which reserved bedaquiline for patients with limited treatment options. DST showed 64.3% of bedaquiline-treated patients with pre-XDR- or XDR-TB, compared with only 14.0% of non-bedaquiline-treated patients. Patients treated with bedaquiline were also more likely to have relapsed or to have already failed on second-line treatment. We note in this context that bedaquiline has been recommended for all patients with MDR-TB or RR-TB in South Korea since December 2020 [27]. In addition, we note that the rate of loss to follow-up was higher in this registry than elsewhere [22]. Results from a much larger (> 3000 patients) retrospective South African registry [28] showed increased rates of treatment success and reduced mortality in patients receiving bedaquiline.

There were higher incidences of treatment-emergent peripheral neuropathy (13.6% vs. 2.4%) and paraesthesia (17.0% vs. 8.3%) in patients receiving bedaquiline. However, most bedaquiline-treated patients with these TEAEs were also receiving linezolid which is associated with neurological or ophthalmological AEs (including optical and peripheral neuropathy) [29]. Moreover, unlike the compulsory reporting required for bedaquiline-treated patients, reporting of serious TEAEs (including fatal events) for non-bedaquiline-treated patients to the Janssen Global Safety Database was not required and therefore may be underreported.

The registry was subject to several limitations. Non-random allocation to treatment and the carefully regulated use of bedaquiline in South Korea would almost inevitably have resulted in selection bias. There was also an imbalance in baseline risk factors between the two treatment groups; however, as the analyses were purely descriptive in nature due to the small sample size, no statistical adjustments for these differences were made. Collection of data as per routine clinical practice is likely to have led to missing data, data collection errors, or incomplete outcome assessment due to loss to follow-up. Moreover, there may have been imbalances in data collection as a result of increased monitoring and reporting for bedaquiline-treated patients. Further, as susceptibility data for bedaquiline were not available, development of resistance could not be evaluated. Lastly, privacy regulations precluded access to the Korean National Health Insurance Service, and long-term data on vital status were therefore not available for patients who were lost to follow-up and whose status was therefore uncertain. Although efforts were made by individual sites to ascertain vital status for these patients, the methodology was not applied consistently across sites.

The data obtained from the present registry should be viewed in relation to other studies of bedaquiline and/or delamanid for MDR-TB in South Korea. A subgroup analysis of 21 Korean MDR-TB patients from the C209 trial showed similar efficacy and safety to the overall C209 population (233 patients), with culture conversion rates at week 24 (end of bedaquiline treatment) of 80.0% and 79.5%, respectively, and 75.0% vs. 72.2% at week 120 [30]. Frequencies and types of AE during treatment were similar in both groups, with a single death which was unrelated to bedaquiline [30]. In another study in 61 patients with MDR-TB treated with bedaquiline (n = 39), delamanid (n = 11), or both for at least 1 month, 55 patients (90.2%) had positive sputum cultures at the start of treatment, and 39 (71%) achieved sputum culture conversion within a median 119 days [31]. Median duration of treatment with bedaquiline or delamanid was 168 days. In a larger national cohort study including 260 patients with MDR-TB/RR-TB, of whom 119 (45.8%) received bedaquiline and 141 (54.2%) delamanid, an overall treatment success rate of 79.2% was reported [22].

Contrasts with other registries and combined analyses are also warranted. Pooled data from five cohorts totalling 537 patients who received bedaquiline in France, Georgia, Armenia, South Africa, and in a further multi-national study showed treatment success rates and mortality of 65.8% and 11.7%, respectively [11]. In a meta-analysis of individual patient data from 50 studies published between 1 January, 2009 and 30 April, 2016, which involved 12,030 patients from 25 countries, 7346 (61%) had treatment success, 1017 (8%) had failure or relapse, and 1729 (14%) died [15]. Treatment including bedaquiline was significantly associated with reduced mortality. Data from a prospective follow-up of 272 South African patients with XDR-TB showed a 24-month favourable outcome rate that was markedly better in patients who received bedaquiline than in those who did not (66.2% vs. 13.2%; p < 0.001); 24-month treatment failure was also less frequent with bedaquiline (5.9% vs. 26.0%; p < 0.001) [9]. Finally, out of 247 culture-confirmed MDR-TB cases completing treatment including bedaquiline in a retrospective observational study across 25 centres in 15 countries, 71.3% achieved treatment success (62.4% cured; 8.9% completed treatment), 13.4% died, 7.3% defaulted, and 7.7% failed [10]. Of interest, and as discussed earlier, WHO data reported in 2021 showed a 59% rate of treatment success for MDR/RR-TB in 2018, the latest patient cohort for which data were available [1].

## Conclusion

These findings are consistent with previous data and support the benefit/risk balance and continued implementation of bedaquiline therapy as part of combination treatment in patients with MDR-TB.

## Supplementary Information

**Additional file 1.** South Korean Treatment Guidelines and supplementary tables.

**Additional file 2.** **Appendix 1**. List of Institutional Review Boards and Ethics Committees.

## Data Availability

The data sharing policy of Janssen Pharmaceutical Companies of Johnson & Johnson is available at https://www.janssen.com/clinical-trials/transparency. As noted on this site, requests for access to the study data can be submitted through Yale Open Data Access (YODA) Project site at http://yoda.yale.edu

## Abbreviations

AEs: Adverse events
AESIs: AEs of special interest
CI: Confidence interval
DST: Drug susceptibility testing
ECG: Eletrocardiogram
eCRF: Electronic clinical report form
FDA: US Food and Drug Agency
KCDC: Korean Centers for Disease Control and Prevention
MCR: Multi-country register
MDR: Multi-drug resistant
MedDRA: Medical Dictionary for Regulatory Activities
NTBERC: National TB Expert Review Committee
OC-RDC: Oracle Remote Data Capture
QTcF: QT interval defined with correction using Friderica’s formula
RR: Rifampicin resistant
SD: Standard deviation
SLIs: Second-line injectables
SMQ: Standardised MedDRA query
TB: Tuberculosis
TEAEs: Treatment-emergent AEs
WHO: World Health Organization
XDR-TB: Extensively drug-resistant TB
