# Supplementary material for: A prospective patient registry to monitor safety, effectiveness, and utilisation of bedaquiline in patients with multidrug-resistant tuberculosis in South Korea
Source: BMC Infect Dis. 2023 Jan 9;23:15. doi: 10.1186/s12879-022-07955-6 (PMC9828359; doi:10.1186/s12879-022-07955-6)
Supplement: Supplementary file 1 — Additional file 1. South Korean Treatment Guidelines and supplementary tables. [file 12879_2022_7955_MOESM1_ESM.docx]

**Additional file 1**

**South Korean Treatment Guidelines**

In South Korea, data were collected from patients who started treatment in 2016 to 2018.

The previous WHO MDR-TB treatment guidelines, issued in 2014, classified anti-TB drugs into five groups to be selected from in a step-down manner, from Group 1 to Group 5. Group 1 contained the first-line oral agents (isoniazid [INH], rifampicin [RMP], ethambutol [EMB], pyrazinamide [PZA], rifabutin); Group 2 contained the injectable anti-TB drugs (streptomycin, kanamycin [KM], amikacin [AM], capreomycin [CM]); Group 3 contained the fluoroquinolones (levofloxacin [LFX], moxifloxacin [MFX], gatifloxacin); Group 4 contained the oral bacteriostatic second-line anti-TB drugs (ethionamide [ETO], prothionamide [PTO], cycloserine [CS], terizidone [TRD], para-aminosalicylic acid [PAS]); and Group 5 contained new anti-TB agents (bedaquiline, delamanid, linezolid [LZD], clofazimine [CFZ], amoxicillin/clavulanate, imipenem/cilastatin, meropenem, high-dose INH, thioacetazone, clarithromycin). Group 5 drugs (new anti-TB medications with limited efficacy and/or long-term safety data) were not recommended to be used routinely in MDR-TB treatment. Based on stepwise selection of drugs, a standard MDR-TB regimen consisted of an 8-month intensive phase with KM-LFX-ETO-CS-PZA and a 12-month maintenance phase with LFX-ETO-CS-PZA. The regimen was to be adjusted based on DST results.

The 2016 WHO MDR-TB treatment guidelines re-classified anti-TB agents into four groups (groups A to D; bedaquiline was included in the add-on agents group D2). The South Korean 2017 MDR-TB treatment guidelines, applied in consideration of domestic circumstances, maintained the previous 5-group classification and bedaquiline was in Group 5, with LZD, delamanid, CFZ, imipenem, meropenem, amoxicillin, and high-dose INH. In South Korea, bedaquiline was approved in 2014. The use of bedaquiline was regulated by the ‘Pre-review system for MDR-TB novel drug use’, implemented from September 2016.

The composition of MDR-TB treatment for patients in South Korea during the registry period was based on the five-group classification of MDR-TB drugs derived from the 2014 WHO MDR-TB treatment guidelines for both bedaquiline-treated and non-bedaquiline-treated patients. Bedaquiline treatment was only allowed for patients with limited treatment options.

| **Table S1** Concomitant TB medications besides bedaquiline**:** safety population | | | |
| --- | --- | --- | --- |
| **Parameter, n (%)** | **Bedaquiline N = 88** | **No bedaquiline N = 84** | **Total N = 172** |
| Patients with ≥ 1 other medication besides bedaquiline prescribed for TB | 88 (100) | 84 (100) | 172 (100) |
| Amikacin | 23 (26.1) | 12 (14.3) | 35 (20.3) |
| Aminosalicylate sodium | 1 (1.1) | 1 (1.2) | 2 (1.2) |
| Aminosalicylic acid | 33 (37.5) | 18 (21.4) | 51 (29.7) |
| Amoxicillin w/ clavulanate potassium | 26 (29.5) | 9 (10.7) | 35 (20.3) |
| Clarithromycin | 2 (2.3) | 0 | 2 (1.2) |
| Clofazimine | 30 (34.1) | 8 (9.5) | 38 (22.1) |
| Cycloserine | 68 (77.3) | 74 (88.1) | 142 (82.6) |
| Delamanid | 13 (14.8) | 22 (26.2) | 35 (20.3) |
| Ethambutol | 17 (19.3) | 26 (31.0) | 43 (25.0) |
| Ethionamide* | 0 | 2 (2.4) | 2 (1.2) |
| Imipenem | 6 (6.8) | 1 (1.2) | 7 (4.1) |
| Isoniazid | 8 (9.1) | 21 (25.0) | 29 (16.9) |
| Kanamycin | 40 (45.5) | 59 (70.2) | 99 (57.6) |
| Kombipak II | 0 | 1 (1.2) | 1 (0.6) |
| Levofloxacin | 30 (34.1) | 44 (52.4) | 74 (43.0) |
| Linezolid | 63 (71.6) | 19 (22.6) | 82 (47.7) |
| Meropenem | 12 (13.6) | 2 (2.4) | 14 (8.1) |
| Moxifloxacin | 24 (27.3) | 40 (47.6) | 64 (37.2) |
| Myrin plus | 0 | 2 (2.4) | 2 (1.2) |
| Prothionamide | 62 (70.5) | 75 (89.3) | 137 (79.7) |
| Pyrazinamide | 38 (43.2) | 72 (85.7) | 110 (64.0) |
| Pyridoxine | 2 (2.3) | 1 (1.2) | 3 (1.7) |
| Rifabutin | 3 (3.4) | 5 (6.0) | 8 (4.7) |
| Rifampicin | 2 (2.3) | 15 (17.9) | 17 (9.9) |
| Streptomycin | 8 (9.1) | 11 (13.1) | 19 (11.0) |

TB, tuberculosis; note: ethionamide is not available in South Korea; these two patients were prescribed ethionamide in another country, which was continued to be used in South Korea.

| **Table S2** Bedaquiline-related treatment-emergent adverse events reported in >1 bedaquiline-treated patient: safety population | |
| --- | --- |
| **Parameter, n (%)** | **Bedaquiline  N = 88** |
| Electrocardiogram QT prolonged | 9 (10.2) |
| Diarrhoea | 8 (9.1) |
| Nausea | 8 (9.1) |
| Vomiting | 4 (4.5) |
| Decreased appetite | 4 (4.5) |
| Arthralgia | 4 (4.5) |
| Dizziness | 4 (4.5) |
| Hypophagia | 3 (3.4) |
| Paraesthesia | 3 (3.4) |
| Pruritus | 3 (3.4) |
| Rash | 3 (3.4) |
| Asthenia | 3 (3.4) |
| Abdominal pain upper | 2 (2.3) |
| Dyspepsia | 2 (2.3) |
| Gastrointestinal disorder | 2 (2.3) |
| Increased alanine aminotransferase | 2 (2.3) |
| Anaemia | 2 (2.3) |
| Cough | 2 (2.3) |
| Patients may have had more than one event, but they are counted once. ‘Possible’, ‘Probable’ and ‘Missing’ relationships to bedaquiline treatment were considered as related. | |

**Table S3** Summary of fatal cases

| **Baseline characteristics of fatal cases** | | | | | | | | |  |
| --- | --- | --- | --- | --- | --- | --- | --- | --- | --- |
| No. of patients | | | 13 | | | 3 | |  |  |
| Mean age, years (SD) | | | 67.4 (14.0) | | | 49.0 (24.3) | |  |  |
|  | | |  | | |  | |  |  |
| Male sex | | | 7 (53.8) | | | 1 (33.3) | |  |  |
|  | | |  | | |  | |  |  |
| Resistance category | | |  | | |  | |  |  |
| MDR-TB | | | 10 (76.9) | | | 3 (100) | |  |  |
| XDR-TB | | | 3 (23.1) | | | 0 | |  |  |
| Treatment category | | |  | | |  | |  |  |
| New | | | 5 (38.5) | | | 2 (66.7) | |  |  |
| Relapse | | | 5 (38.5) | | | 0 | |  |  |
| Treatment after failure of 1^st-^line drugs | | | 1 (7.7) | | | 1 (33.3) | |  |  |
| Treatment after failure of 2^nd-^line drugs | | | 2 (15.4) | | | 0 | |  |  |
| **TEAEs leading to death** | | | | | | | | |  |
| Total no. of patients in population | | | 88 | | | 84 | |  |  |
| ≥ 1 TEAE leading to death | | | 12 (13.6) | | | 3 (3.6) | |  |  |
| Pneumonia | | | 5 (5.7) | | | 0 | |  |  |
| Dyspnoea | | | 3 (3.4) | | | 1 (1.2) | |  |  |
| Acute renal failure | | | 1 (1.1) | | | 1 (1.2) | |  |  |
| Acute respiratory distress syndrome | | | 1 (1.1) | | | 0 | |  |  |
| Hypoglycaemia | | | 1 (1.1) | | | 0 | |  |  |
| Metabolic acidosis | | | 1 (1.1) | | | 0 | |  |  |
| Hepatocellular carcinoma | | | 1 (1.1) | | | 0 | |  |  |
| Ureteric cancer | | | 1 (1.1) | | | 0 | |  |  |
| Aortic rupture | | | 1 (1.1) | | | 0 | |  |  |
| Hypotension | | | 1 (1.1) | | | 0 | |  |  |
| Death (cause not reported) | | | 1 (1.1) | | | 0 | |  |  |
| Delirium | | | 1 (1.1) | | | 0 | |  |  |
| Aspiration | | | 0 | | | 1 (1.2) | |  |  |
| Fulminant hepatitis | | | 0 | | | 1 (1.2) | |  |  |
|  | | | | | | | | | |
| **Age/sex: disease type** | **Medical history** | **Fatal event(s)** | | **Reported causality (bedaquiline only)** | | | **Comments** | | |
| **Bedaquiline treatment** | | | | | | | | | |
| 91/F: XDR-TB | Diabetes, ischemic heart disease, hypertension, hypokalaemia, delirium, pleural effusion | Delirium, pneumonia, hypoglycaemia, hypotension | | Not related | | | Sputum culture positive at time of fatal event. **TB reported as cause of death** | | |
| 61/M: MDR-TB | Ureter cancer, benign prostatic hypertension | Worsening of ureter cancer | | Not related | | | **Worsening of ureter cancer reported as cause of death** | | |
| 88/F: MDR-TB | Diabetes, hypertension | Pneumonia, acute renal failure | | Not related | | | Prior history of pneumonia | | |
| 71/M: XDR-TB | HCC, diabetes, cough, hypertension | Worsening of HCC | | Not related | | | **Worsening of pre-existing HCC reported as cause of death** | | |
| 69/F: XDR-TB | Lung cancer, COPD, leukopenia, peripheral neuropathy | Pneumonia, Dyspnoea | | Not related | | | Worsening of small cell lung cancer before experiencing pneumonia. | | |
| 79/M: XDR-TB | COPD | Death | | Not reported | | | Bedaquiline started and stopped several times for unspecified reasons. **Cause of death was not reported** | | |
| 74/F: XDR-TB | Anaemia, hypertension, osteoporosis, adrenal insufficiency | Aortic rupture | | Not related | | | Limited fatal event information available | | |
| 51/F: MDR-TB | Peritoneal adenocarcinoma, dyspepsia, depression, anticoagulation, constipation | Dyspnoea | | Not related | | | **Cause of death: malignant neoplasm of appendix vermiformis** | | |
| 54/M: MDR-TB | Alcoholic hepatic cirrhosis, type 2 diabetes | Pneumonia | | Not related | | | Fall and haematoma causing hospitalisation approximately 5 weeks before death | | |
| 56/M: MDR-TB | Gout, gastritis | Dyspnoea | | Not related | | | Severe (nonfatal) haemoptysis, bile duct stone, enterococcal infection also reported | | |
| 57/M: MDR-TB | Alcohol abuse, diabetes, gout | Acute respiratory disorder | | Not related | | | Severe (nonfatal) sepsis and acute renal failure also reported | | |
| 76/F: MDR-TB | Pneumonia, chronic kidney disease, diabetes, hypothyroidism | Pneumonia, metabolic acidosis | | Not related | | | Treatment ongoing for concurrent pneumonia at start of bedaquiline treatment | | |
| 47/M: MDR-TB | Nausea | Myelopathy^a^ | | Not related | | | Patient died approximately 3 months after stopping MDR-TB treatment | | |
| **No bedaquiline treatment** | | | | | | | | | |
| 71/M: MDR-TB | Type 1 diabetes, heart disease, heart rhythm disorder, acute brain embolic infarct, recurrent embolic infarction, adrenal insufficiency, chronic kidney disease, hypertension | Aspiration | | | Concurrent acute coronary syndrome, pyrexia, pneumonia, urinary tract infection, increased AST/ALT, acute renal failure, chronic renal failure, decubitus ulcer | | | | |
| 53/F: MDR-TB | Depression, type 2 diabetes, heart disease, hypothyroidism, idiopathic pulmonary fibrosis, interstitial lung disease, unspecified pain | Dyspnoea | | | Dyspnoea onset 4.5 months before death. Patient was receiving MDR-TB medications up to 2 days prior to death. **Cause of death reported as pneumonia** | | | | |
| 23/M: MDR-TB |  | Fulminant hepatitis; acute renal failure | | | Patient receiving MDR-TB medications up to 2 weeks prior to death; these were stopped due to fulminant hepatitis and acute renal failure. **Cause of death reported as TB** | | | | |
| ALT, alanine transaminase; AST, aspartate transaminase; COPD, chronic obstructive pulmonary disease; F, female; HCC, hepatocellular carcinoma; M, male; MDR, multidrug-resistant; SD, standard deviation; TB, tuberculosis; TEAE, treatment-emergent adverse event; XDR, extensively drug-resistant. | | | | | | | | | |
