# Supplementary material for: A prospective patient registry to monitor safety, effectiveness, and utilisation of bedaquiline in patients with multidrug-resistant tuberculosis in South Korea
Source: BMC Infect Dis. 2023 Jan 9;23:15. doi: 10.1186/s12879-022-07955-6 (PMC9828359; doi:10.1186/s12879-022-07955-6)
Supplement: Supplementary file 2 — Additional file 2. Appendix 1. List of Institutional Review Boards and Ethics Committees. [file 12879_2022_7955_MOESM2_ESM.docx]

| TMC207TBC4002 | South Korea | Kwangju Christian Hospital Institutional Review Board/  37, Yangnim-ro, Nam-gu,  Gwangju, 61661,  Republic of Korea | 202  Kwangju Christian Hospital  37, Yangnim-ro, Nam-gu,  Gwangju, 61661,  Republic of Korea | Site, Local: 02Mar2016 |
| --- | --- | --- | --- | --- |
|  | South Korea | Asan Medical Center Institution Review Board  Convergence Innovation Bldg., 88, Olympic-ro 43-gil, Songpa-gu, 05505 Seoul  Republic of Korea | 203  Asan Medical Center  5505 Seoul  Republic of Korea | Site, Local: 28.03.2016 |
|  | South Korea | Institutional Review Board for Human Research Yonsei University Wonju Severance Christian Hospital/  20, Ilsan-ro,  Wonju-si, Gangwon-do, 26426,  Republic of Korea | 204  Won-Yeon Lee  Wonju Severance Christian Hospital  20, Ilsan-ro,  Wonju-si, Gangwon-do, 26426,  Republic of Korea | Site, Local: 21Jun2016 |
|  | South Korea | Seobuk Hospital Institutional Review Board /  49, Galhyeon-ro 7-gil, Eunpyeong-gu, Seoul, 03433,  Republic of Korea | 205  Hae Sook Seo  Seobuk Hospital  49, Galhyeon-ro 7-gil, Eunpyeong-gu, Seoul, 03433,  Republic of Korea | Site, Local: 01Jun2016 |
|  | South Korea | National Medical Center IRB  Clinical Trial Ceneter, 245, Eulji-ro, Jung-gu  04564 Seoul  Republic of Korea | 206  National Medical Center  245, Eulji-ro, Jung-gu  4564 Seoul  Republic of Korea | Site, Local: 28.01.2016 |
|  | South Korea | Chung-Ang University Hospital IRB  B2, Chung-Ang Bldg., 102, Heukseok-ro, Dongjak-gu  06973 Seoul  Republic of Korea | 207  Chung-Ang University Hospital  102, Heukseok-ro, Dongjak-gu  6973 Seoul  Republic of Korea | Site, Local: 24.06.2016 |
|  | South Korea | Pusan National University Hospital IRB  3F, Convergence Medical Research Center, 179, Gudeok-ro, Seo-gu  Busan, 49241  Republic of Korea | 208  Pusan National University Hospital  179, Gudeok-ro, Seo-gu  49241 Busan  Republic of Korea | Site, Local: 25.12.2015 |
|  | South Korea | Institutional Review Board for Human Research Ulsan University Hospital/  877, Bangeojinsunhwando-ro, Dong-gu, Ulsan, 44033,  Republic of Korea | 209  Taehoon Lee  Ulsan University Hospital  877, Bangeojinsunhwando-ro, Dong-gu, Ulsan, 44033,  Republic of Korea | Site, Local: 22Jan2016 |
|  | South Korea | Incheon Medical Center IRB  3F, Main Bldg., 217 Bangchuk-ro, Dong-gu  Incheon 22532  Republic of Korea | 211  Incheon Medical Center  22532 Incheon  Republic of Korea | Site, Local: 30.12.2015 |
|  | South Korea | Chonnam National University Hospital Institution Review Board  5F, 3 dong, 42 Jebong-ro, Dong-gu  61469 Gwangju  Republic of Korea | 212  Chonnam National University Hospital  42, Jebong-ro, Dong-gu  61469 Gwangju  Republic of Korea | Site, Local: 26.01.2016 |
|  | South Korea | Korea University Ansan Hospital Institutional Review Board/  123, Jeokgeum-ro, Danwon-gu,  Ansan-si, Gyeonggi-do, 15355,  Republic of Korea | 214  Seung Heon Lee  Korea University Ansan Hospital  123, Jeokgeum-ro, Danwon-gu,  Ansan-si, Gyeonggi-do, 15355,  Republic of Korea | Site, Local: 12Apr2016 |
|  | South Korea | Institutional Review Board of Ajou University hospital /  164, World cup-ro, Yeongtong-gu,  Suwon-si, Gyeonggi-do, 16499,  Republic of Korea | 215  Kwang Joo Park  Ajou University Medical Center  164, World cup-ro, Yeongtong-gu,  Suwon-si, Gyeonggi-do, 16499,  Republic of Korea | Site, Local: 28Apr2016 |
|  | South Korea | Institutional Review Board of Incheon St. Mary's Hospital, The Catholic University of Korea/  56, Dongsu-ro, Bupyeong-gu,  Incheon, 21431,  Republic of Korea | 216  Ju Sang Kim  The Catholic University of Korea Incheon St. Mary's Hospital  56, Dongsu-ro, Bupyeong-gu,  Incheon, 21431,  Republic of Korea | Site, Local: 13Apr2016 |
|  | South Korea | Dankook University Hospital IRB  3F, Somang ldg., 185, Manghyang-ro, Dongnam-gu, Cheonan-si  31116 Chungcheongnam-do  Republic of Korea | 217  Dankook University Hospital  201 Manghyang-ro, Dongnam-gu  31116 Cheonan-si  Republic of Korea | Site, Local: 26.04.2016 |
|  | South Korea | Institutional Review Board of Seoul St. Mary's Hospital, The Catholic University of Korea/  222, Banpo-daero, Seocho-gu, Seoul,  06591,  Republic of Korea | 218  Seung Joon Kim  The Catholic University of Korea Seoul St. Mary’s Hospital  222, Banpo-daero, Seocho-gu, Seoul,  06591,  Republic of Korea | Site, Local: 02Aug2016 |
|  | South Korea | Institutional Review Board of Yeungnam University Medical Center/  170, Hyeonchung-ro, Namgu,  Daegu, 42415,  Republic of Korea | 219  Eun Young Choi  Yeungnam University Medical Center  170, Hyeonchung-ro, Namgu,  Daegu, 42415,  Republic of Korea | Site, Local: 14Jul2016 |
|  | South Korea | Yonsei University College of Medicine IRB  50-1, Yonsei-ro,  Seodaemun-gu  03722 Seoul  Republic of Korea | 220  Yonsei University Health System - Severance Hospital  50, Yonsei-ro,  Seodaemun-gu  3722 Seoul  Republic of Korea | Site, Local: 23.07.2016 |
|  | South Korea | Institutional Review Board of Uijeongbu St. Mary's Hospital, The Catholic University of Korea/  271, Cheonbo-ro,  Uijeongbu-si, Gyeonggi-do, 11765  Republic of Korea | 221  Jin Woo Kim  271, Cheonbo-ro,  Uijeongbu-si, Gyeonggi-do, 11765  Republic of Korea | Site, Local: 15Mar2018 |
|  | South Korea | Korea University Guro Hospital IRB  148, Gurodong, Gurogu  03722 Seoul  Republic of Korea | 222  Korea University Guro Hospital  148, Gurodong-ro  8308 Guro -gu  Republic of Korea | Site, Local: 23.07.2016 |
